# Supplementary material for: The Association between Ambient PM2.5 and Low Birth Weight in California
Source: Int J Environ Res Public Health. 2022 Oct 19;19(20):13554. doi: 10.3390/ijerph192013554 (PMC9602828; doi:10.3390/ijerph192013554)
Supplement: Supplementary file 1 [file ijerph-19-13554-s001.zip › ijerph-1954950-supplementary.pdf]

Table S1. Percent change in low birth weight per 1 μg/m^3 change in $PM_{2.5}$ stratified by median race/ethnicity in California census tracts.

**Race/Ethnicity**	**≤ Median (n = 3893)** *β (95% CI)*	**> Median (n=3892)** *β (95% CI)*
Hispanic	0.04 (0.02, 0.06)	0.02 (0.00, 0.04)
White	0.01 (-0.01, 0.03)	0.04 (0.02, 0.06)
African American	0.03 (0.02, 0.05)	0.01 (-0.01, 0.03)
Asian American	0.02 (0.00, 0.03)	0.03 (0.01, 0.06)

All models adjusted for the other race/ethnicity groups, poverty and toxic releases from facilities. Median % Hispanic: 31.4%, Median % White: 37.1%, Median % African American: 2.6%, Median % Asian American: 8.2%.
